# CXCL16/CXCR6 is involved in LPS‐induced acute lung injury via P38 signalling

**DOI:** 10.1111/jcmm.14419

**Published:** 2019-06-14

**Authors:** Guo‐wei Tu, Min‐jie Ju, Yi‐jun Zheng, Guang‐wei Hao, Guo‐guang Ma, Jun‐yi Hou, Xue‐peng Zhang, Zhe Luo, Li‐ming Lu

**Affiliations:** ^1^ Department of Critical Care Medicine, Zhongshan Hospital Fudan University Shanghai China; ^2^ Department of Critical Care Medicine, Xiamen Branch, Zhongshan Hospital Fudan University Xiamen China; ^3^ Shanghai Institute of Immunology Shanghai Jiaotong University School of Medicine Shanghai China

**Keywords:** 16HBE, acute lung injury, CXCL16, NF‐κB, p38 signal

## Abstract

Although several chemokines play key roles in the pathogenesis of acute lung injury (ALI), the roles of chemokine (C‐X‐C motif) ligand 16 (CXCL16) and its receptor C‐X‐C chemokine receptor type 6 (CXCR6) in ALI pathogenesis remain to be elucidated. The mRNA and protein expression of CXCL16 and CXCR6 was detected after lipopolysaccharide (LPS) stimulation with or without treatment with the nuclear factor‐κB (NF‐κB) inhibitor pyrrolidine dithiocarbamate (PDTC). Lung injury induced by LPS was evaluated in CXCR6 knockout mice. CXCL16 level was elevated in the serum of ALI patients (n = 20) compared with healthy controls (n = 30). CXCL16 treatment (50, 100, and 200 ng/mL) in 16HBE cells significantly decreased the epithelial barrier integrity and E‐cadherin expression, and increased CXCR6 expression, reactive oxygen species (ROS) production, and p38 phosphorylation. Knockdown of CXCR6 or treatment with the p38 inhibitor SB203580 abolished the effects of CXCL16. Moreover, treatment of 16HBE cells with LPS (5, 10, 20 and 50 μg/mL) significantly increased CXCL16 release as well as the mRNA and protein levels of CXCL16 and CXCR6. The effects of LPS treatment (20 μg/mL) were abolished by treatment with PDTC. The results of the luciferase assay further demonstrated that PDTC treatment markedly inhibited the activity of the CXCL16 promoter. In conclusion, CXCL16, whose transcription was enhanced by LPS, may be involved in ROS production, epithelial barrier dysfunction and E‐cadherin down‐regulation via p38 signalling, thus contributing to the pathogenesis of ALI. Importantly, CXCR6 knockout or inhibition of p38 signalling may protect mice from LPS‐induced lung injury by decreasing E‐cadherin expression.

## 1 INTRODUCTION

Acute lung injury (ALI) or acute respiratory distress syndrome (ARDS), the more severe form of ALI, is characterized by diffuse pulmonary infiltration, increased vascular permeability, impaired gas exchange, decreased pulmonary compliance, pulmonary oedema and ultimately respiratory failure.^1^ ALI/ARDS is the leading cause of in‐hospital mortality in clinical pulmonology^2^ and results from the complex responses of the lung to a various direct (eg, pneumonia, inhalational injury and gastric aspiration) and indirect (eg, sepsis, pancreatitis and multiple blood transfusion) insults.^3^ Increasing evidence has suggested the involvement of oxidative stress in the pathogenesis of ALI/ARDS.^4^ Patients with ARDS have increased levels of hydrogen peroxide in breath condensate^5^, ^6^ and an excess of oxidatively modified proteins in bronchoalveolar lavage (BAL).^7^, ^8^, ^9^

Chemokines, a large family of chemoattractant cytokines, play key roles in the immune system through binding to Gprotein‐coupled serpentine receptors.^10^ Evidence from clinical studies and animal models of ALI has supported the important role of chemokines in the pathogenesis of ALI. It has been reported that C‐C motif chemokine ligand 2 (CCL2), chemokine (C‐X‐C motif) ligand 1 (CXCL1), CXCL5 and CXCL8 are elevated in the plasma and BAL of patients with ALI.^11^, ^12^ In animal models of ALI, chemokine levels in the lungs (eg, CXCL1, CXCL2 and CXCL5) are raised, and inhibitors of chemokines^13^, ^14^, ^15^, ^16^ or their receptors^17^ can mitigate injury. The expression of CXCL16, expressed in soluble and transmembrane forms, has been observed in a wide range of cell types, including inflammatory cells (eg, macrophages, dendritic cells and monocytes)^18^, ^19^ and non‐inflammatory cells (eg, human bronchial epithelial cells^20^ and renal podocytes^21^). A previous study suggested that CXCL16 and its receptor C‐X‐C chemokine receptor type 6 (CXCR6) are implicated in the tumourigenesis and metastasis of lung cancer.^22^ However, it is not yet known whether CXCL16/CXCR6is involved in the pathogenesis of ALI. p38 mitogen‐activated protein kinases play critical roles in mediating cellular responses to stressors, and evidence has suggested that p38 is involved in the development of different stimuli‐induced ALI/ARDS.^23^, ^24^, ^25^ Nuclear factor‐kappa B (NF‐κB), a critical transcription factor that regulates the expression of various cytokines and chemokines, plays an important role in ALI.^26^ However, it remains unclear whether p38 and NF‐κB are associated with CXCL16/CXCR6 during ALI progression.

Bronchial epithelial cells form a continuous, highly regulated airway barrier that prevents the inhalation of environmental factors such as airborne allergens, contaminants and pathogens.^27^ Evidence has indicated that inflammatory insult‐induced disruption of the airway epithelial barrier is involved in the pathogenesis of ALI. E‐cadherin, a well‐studied member of the classical cadherin family, is localized to the cell‐cell adhesion junctions (AJs) of epithelial cells and plays a critical role in maintaining cell polarity and the normal epithelial structure.^28^, ^29^, ^30^ Loss of E‐cadherin expression has been observed in lung diseases such as asthma and chronic obstructive pulmonary disease.^29^

Lipopolysacchride (LPS), a glycolipid isolated from Gram‐negative bacteria, may trigger lung inflammation, which induces the accumulation of neutrophils, production of reactive oxygen species (ROS), and secretion of cytokines, ultimately leading to ALI/ARDS.^31^ In the present study, we explored the effects of CXCL16 on barrier integrity and ROS production in the 16HBE human bronchial epithelial cell line. We then used an LPS‐treated cell model and well‐established animal model to explore the role of CXCL16/CXCR6 in the pathogenesis of ALI.^32^, ^33^

## 2 MATERIALS AND METHODS

### 2.1 Serum samples

Twenty patients (12 males and 8 females) with ALI as defined by the criteria of the North American European Consensus Conference^1^ were enrolled in this study. The median age was 58 (44‐65) years. All the patients were diagnosed as septic shock complicated with ALI. The serum samples of the patients were collected at the admission of intensive care unit (ICU). Thirty age‐matched healthy volunteers served as control samples. Serum samples were stored at −80°C until use. This study was approved by the Ethics Committee of Zhongshan Hospital, Fudan University, Shanghai, China. All animal experiments were performed in accordance with established guidelines for the care and use of laboratory animals.

### 2.2 Enzyme‐linked immunosorbent assay

CXCL16 levels in the serum and cell supernatant were determined with an enzyme‐linked immunosorbent assay (ELISA)kit (Xinyu Biotechnology, Shanghai, China) following the manufacturer's instructions.

### 2.3 Cell culture

The human bronchial epithelial cell line 16HBE^34^, ^35^ was obtained from the Cell Bank of Chinese Academy of Sciences (Shanghai, China). Cells were cultured in RPMI‐1640 medium (Hyclone, Logan, UT) supplemented with 10% foetal bovine serum (GibcoBRL, Carlsbad, CA) and antibiotics at 37°C in 5% CO_2_.

### 2.4 RNA interference

The human CXCR6 small interfering RNA (siRNA) oligonucleotides (siCXCR6‐1, 5‐GCAGUUCAGCAAGGUCUUUUU‐3; siCXCR6‐2, 5‐GCAUCACUGUGGAUCGUUUUU‐3; siCXCR6‐3, 5‐GCAGCACACACUGGGAAUAUU‐3) and non‐silencing siRNA (siNC) were purchased from GenePharma (Shanghai, China). 16HBE cells were transfected with CXCR6 siRNA or siNC using Lipofectamine2000 (Invitrogen, Carlsbad, CA) according to the manufacturer's protocol. After 48 hours, the knockdown efficiency of siRNA was assessed by western blot analysis.

### 2.5 Cell treatment

To study the effects of CXCL16, 16HBE cells were treated with CXCL16 (PeproTech, Rocky Hill, NJ) at doses of 0, 50, 100 and 200 ng/mL. At 0 hour before and 24 hours after CXCL16 treatment, epithelial permeability was measured using fluorescein isothiocyanate (FITC)‐conjugated dextran. After 24 hours of treatment, ROS production and protein expression were detected by dichlorodihydrofluorescein diacetate (DCFH‐DA) and western blot analysis, respectively.

To study the involvement of CXCR6 and p38, the 16HBE cells were divided into the following four groups: Group 1, siNC; Group 2, siNC + CXCL16; Group 3, siCXCR6 + CXCL16 and Group 4, siNC + SB+CXCL16. The cells in Group 3 were transfected with CXCR6 siRNA (siCXCR6), whereas cells in the other groups were transfected with control siRNA (siNC). At 24 hours after transfection, cells in Groups 2 and 3 were exposed to 100 ng/mL CXCL16, whereas cells in Group 4 were exposed to 1 μmol/L SB203580 (Selleck, Houston, TX) and 100 ng/mL CXCL16. Epithelial permeability was measured at 0 hour before and 24 hours after CXCL16 treatment, and protein expression was detected at 24 hours after CXCL16 treatment.

To determine the effects of LPS on CXCL16/CXCR6, 16HBE cells were treated with LPS (Sigma‐Aldrich, St. Louis, MO) at doses of 0, 5, 10, 20 and 50 μg/mL for 24 hours. The mRNA and protein expression of CXCL16 and CXCR6 were evaluated by quantitative real‐time PCR and western blotting, respectively. To investigate the involvement of NF‐κB, 16HBE cells were exposed to LPS (20 μg/mL) and the NF‐κB inhibitor pyrrolidine dithiocarbamate (PDTC) (10 μmol/L; Sigma‐Aldrich) for 24 hours.

### 2.6 Measurement of epithelial permeability

16HBE cells (1 × 10^4^ cells per well) were seeded on Transwell inserts. After culturing for about 72 hours, the cells formed cell monolayers and CXCL16 (0, 50, 100 and 200 ng/mL) was added to the inserts. At 0 hour before and 24 hours after CXCL16 treatment, FITC‐conjugated dextran (4 kDa, 1 mg/mL; Sigma‐Aldrich) was added to the apical compartment. After incubation at 37°C for 2 hours, aliquots of the basal compartments were collected and the fluorescence was measured at an emission wavelength of 520 nm and excitation wavelength of 492 nm. The changes in FITC‐dextran flux were calculated, and the mean values of the control group were set at 100%.

### 2.7 Measurement of intracellular ROS

Production of intracellular ROS was measured using DCFH‐DA (Beyotime, Shanghai, China) in accordance with the manufacturer's instructions. In brief, the cells were trypsinized, incubated with 10 µmol/L DCFH‐DA at 37°C for 20 minutes, rinsed with RPMI‐1640 and analysed by flow cytometry (BD Biosciences, Franklin Lakes, NJ) at an excitation wavelength of 480 nm and emission wavelength of 525 nm.

### 2.8 Protein extraction and western blot analysis

For total protein extraction, the cells were lysed in RIPA buffer (Solarbio, Beijing, China) supplemented with proteinase inhibitor cocktail (Sigma‐Aldrich) on ice for 30 minutes. The lysate was cleared by centrifugation at 10 000 *g* for 20 minutes. Cytosol and nuclear proteins were extracted using NE‐PE Nuclear and Cytoplasmic Extraction Reagents (Thermo Fisher Scientific, Rockford, IL) according to the manufacturer's protocol.

For western blotting, the lysate (30 μg protein per sample) was separated by sodium dodecyl sulphate polyacrylamide gel electrophoresis and transferred onto a nitrocellulose membrane (Millipore, Bradford, MA). Non‐specific binding was blocked by incubation in 5% skim milk for 1 hour at room temperature. Then, the membranes were incubated overnight at 4°C with the following antibodies: anti‐CXCL16 (Cat. #Ab101404; Abcam, Cambridge, MA), anti‐CXCR6 (Cat. #ab8023; Abcam), anti‐phosphorylated (p)‐p38 (Cat. #9211; Cell Signaling Technology, Danvers, MA), anti‐p38 (Cat. #9212; Cell Signaling Technology), anti‐E‐cadherin (Cat. #14472; Cell Signaling Technology), anti‐GAPDH (Cat. #5174; Cell Signaling Technology), anti‐NF‐κB p65 (Cat. #8242; Cell Signaling Technology), anti‐H3 (Cat. #4499s; Cell Signaling Technology), and anti‐β‐actin (Cat. #4970; Cell Signaling Technology). After washing three times with Tris‐buffered saline with Tween 20, the membranes were incubated with horseradish peroxidase‐conjugated secondary antibody for 1 hour at room temperature. Bands were detected by chemiluminescence using ECL Western Blotting Substrate (Millipore) and quantified by Image J software. All western blot analyses were repeated at least three times.

### 2.9 RNA extraction and quantitative real‐time PCR

Total RNA was extracted using TRIzol Reagent (Invitrogen) and reverse transcription was performed using the RevertAid First‐Strand cDNA Synthesis Kit according to the manufacturer's protocols. Real‐time PCR was performed using the SYBR®GreenKit (Thermo Scientific) on the ABI 7300 Fast Real‐Time PCR System (Applied Biosystems, Foster City, CA). The primer sequences are listed in Table 1.

**Table 1:** Primers used for real‐time PCR

Gene	Primer sequences	Product size
CXCL16	Forward: 5'‐CCCGCCATCGGTTCAGTTC‐3'; Reverse: 5'‐CCCCGAGTAAGCATGTCCAC‐3'	181 bp
CXCR6	Forward: 5'‐GACTATGGGTTCAGCAGTTTCA‐3'; Reverse: 5'‐GGCTCTGCAACTTATGGTAGAAG‐3'	169 bp
GAPDH	Forward: 5'‐CACCCACTCCTCCACCTTTG‐3'; Reverse: 5'‐CCACCACCCTGTTGCTGTAG‐3'	110 bp

### 2.10 Luciferase assay

The CXCL16 promoter was inserted into the pGL3 Vector (Promega, Madison, WI) to construct the pGL3‐CXCL16‐p plasmid. 16HBE cells were co‐transfected with pRL‐CMV plasmid (Promega) and pGL3‐CXCL16‐p using Lipofectamine 2000 reagent (Invitrogen). After 6 hours, the cells were treated with PDTC or DMSO. Luciferase assays (Dual‐Luciferase Reporter Assay System, Promega) were performed at 24 hours post‐transfection. The luciferase activity was normalized to the activity of Renilla luciferase.

### 2.11 Animals and lung inflammatory injury

CXCR6^‐/‐^ C57BL/6 mice were a gift from Dr Shijun Wang (Shanghai Institute of Cardiovascular Diseases, Zhongshan Hospital, Fudan University).These mice were constructed by CyagenBiosciences (Suzhou, China) with CRISPR/Cas‐mediated genome engineering. Eight‐week‐old male CXCR6^‐/‐^ mice and wild‐type (WT) C57BL/6 mice were used. Experimental protocols were approved by the Institutional Animal Care and Use Committee at Zhongshan Hospital, Fudan University. Lung injury model was established by intratracheal injection with LPS (Sigma‐Aldrich) under anaesthetization with ether. Mice in the WT/control group received 0.9% NaCl. The mice in the WT/LPS + SB group underwent intraperitoneal injection of SB203580 (5 mg/kg) and intratracheal injection of LPS as described above. At 24 hours after LPS injection, mice from each group were euthanized and lung tissues were collected. The survival rates of the mice (n = 12) were recorded for 7 days.

### 2.12 Haematoxylin and eosin and immunohistochemical staining

The collected lung specimens were fixed overnight in 10% neutral formalin solution, followed by dehydration, clearing, paraffin embedding. Then they were cut into 5‐μm thick serial sections. Pathological analysis was performed on sections stained with haematoxylin and eosin (HE). The expression and distribution of E‐cadherin was observed in sections immunohistochemically stained with anti‐E‐cadherin mouse monoclonal antibody (Cat. #Ab76055; Abcam).

### 2.13 Statistical analysis

The results are presented as the mean ± standard deviation of three independent experiments. Statistical significance was determined with Statistical Package for the Social Sciences software version 16.0 (SPSS, Inc, Chicago, IL). Student's *t* test or one‐way analysis of variance combined with post‐hoc Turkey's analysis was used to evaluate the data. *P* values less than 0.05 were considered statistically significant.

## 3 RESULTS

### 3.1 Serum levels of CXCL16 were elevated in ALI patients

**Figure 1:**
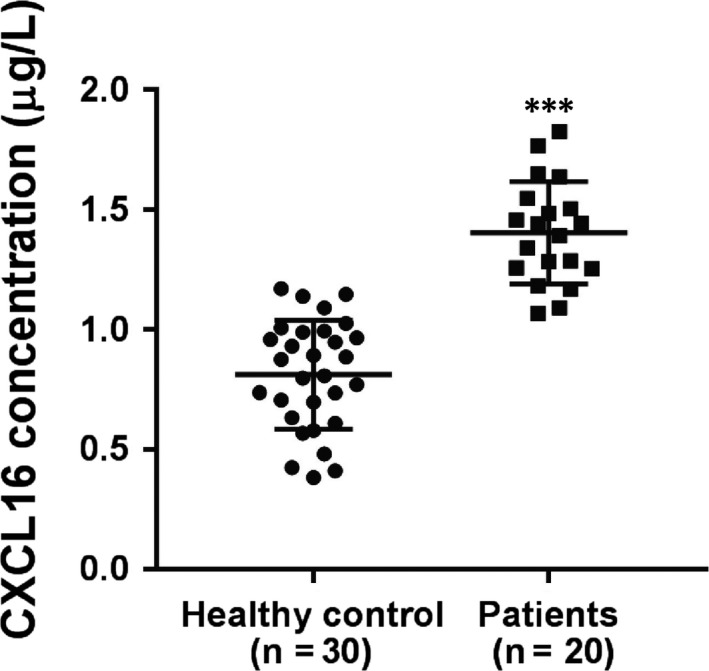
Serum levels of CXCL16. Serum levels of CXCL16 were elevated in ALI patients as demonstrated by ELISA (***P < 0.001 vs healthy controls)

To determine whether CXCL16 is involved in the pathogenesis of ALI, serum levels of CXCL16 in 30 healthy volunteers and 20 patients with ALI were detected by ELISA. As shown in Figure 1, serum levels of CXCL16 were higher in ALI patients (1.40 ± 0.05 μg/L) than in healthy controls (0.81 ± 0.04 μg/L) (*P* < 0.001).

### 3.2 CXCL16‐induced epithelial barrier dysfunction and ROS production

**Figure 2:**
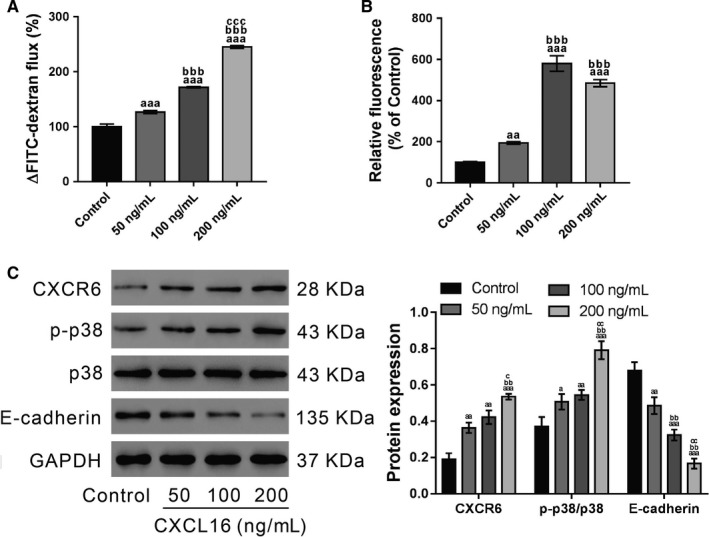
Effects of CXCL16 on epithelial barrier integrity, ROS production and p38 phosphorylation. 16HBE cells were exposed to CXCL16 (50, 100 and 200 ng/mL), and control cells did not receive any treatment. A, Epithelial permeability was measured by using FITC‐conjugated dextran at 0 h before and 24 h after CXCL16 treatment. B, ROS production was determined by DCFH‐DA staining and flow cytometry analysis at 24 h after CXCL16 treatment. C, The levels of CXCR6, p‐p38, p38 and E‐cadherin were detected by western blotting at 24 h after CXCL16 treatment. Representative blots and quantification from three experiments are shown (^a^ *P* < 0.05, ^aa^ *P* < 0.01, ^aaa^ *P* < 0.001 vs control cells; ^bb^ *P* < 0.01, ^bbb^ *P* < 0.001 vs cells treated with 50 ng/mL CXCL16; ^c^ *P* < 0.05, ^cc^ *P* < 0.01, ^ccc^ *P* < 0.001 vs cells treated with 100 ng/mL CXCL16)

To determine if CXCL16 affects airway epithelial barrier integrity, epithelial permeability was measured by FITC‐dextran before CXCL16 treatment (50, 100 and 200 ng/mL) and 24 hours after treatment. The results showed that CXCL16 increased cell permeability in a dose‐dependent manner (Figure 2A). Considering that excessive ROS can mediate the disruption of cellular AJs,^36^, ^37^, ^38^ ROS production was determined in the 16HBE cells. As demonstrated by DCF‐DA staining followed by flow cytometry, exposure to CXCL16 for 24 hours significantly promoted ROS production, and the dose of 100 ng/mL had the most obvious effects (Figure 2B).

Then, the expression of CXCR6 (CXCL16 receptor), E‐cadherin (key component of AJs) and p‐p38 was detected by western blotting. As demonstrated in Figure 2C, CXCL16 exposure significantly enhanced the levels of p‐p38 and CXCR6, but inhibited E‐cadherin expression.

### 3.3 CXCR6 and p38 signalling mediates the effects of CXCL16 on epithelial barrier dysfunction

**Figure 3:**
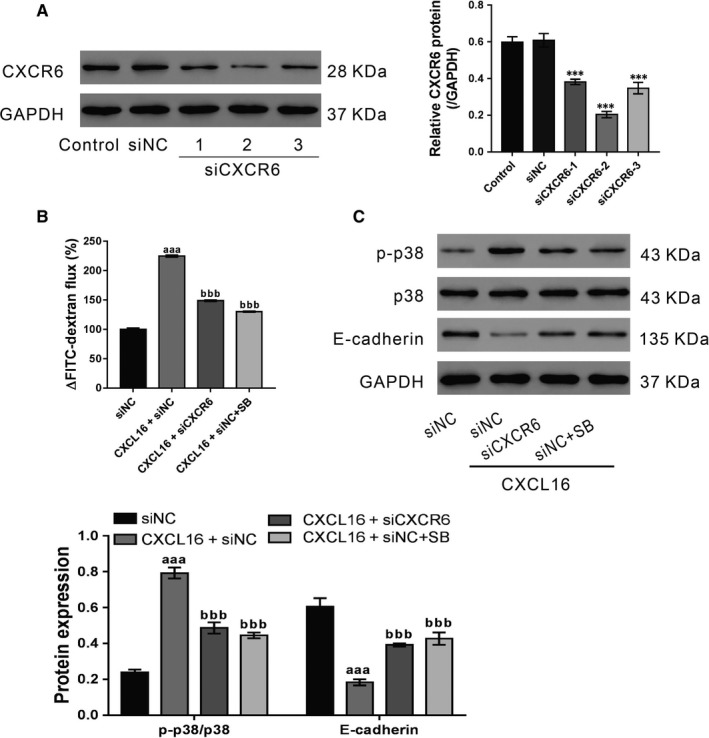
CXCR6 and p38 signalling mediate the effects of CXCL16 on epithelial barrier dysfunction. A, 16HBE cells were transfected with CXCR6 siRNA (siCXCR6‐1, 2, or 3) or control siRNA (siNC) and CXCR6 expression was determined after 48 h. Representative blots and quantification from three experiments are shown (***P < 0.001 vs siNC cells). B and C, 16HBE cells were divided into four groups: Group 1, siNC; Group 2, siNC + CXCL16; Group 3, siCXCR6 + CXCL16; Group 4, siNC + SB+CXCL16. The cells in Group 3 were transfected with CXCR6 siRNA (siCXCR6), whereas cells in the other groups were transfected with control siRNA (siNC). At 24 h post‐transfection, cells in Group 2 and 3 were treated with 100 ng/mL CXCL16, whereas cells in Group 4 were treated with 1 μmol/L SB203580 and 100 ng/mL CXCL16. Epithelial permeability was measured at 0 h before and 24 h after CXCL16 treatment (B). The levels of CXCR6, p‐p38, p38, and E‐cadherin were detected at 24 h after CXCL16 treatment (C). Representative blots and quantification from three experiments are shown (^aaa^ *P* < 0.001 vs siNC; ^bbb^ *P* < 0.001 vs CXCL16 + siNC)

To investigate the involvement of CXCR6 in CXCL16‐stimulated epithelial barrier dysfunction, CXCR6 expression in 16HBE cells was knocked down by siRNA transfection. Immunoblot analysis showed that all three CXCR6 siRNAs (siCXCR6) caused significant knockdown of CXCR6 compared with the control siRNA (siNC) (Figure 3A). At 24 hours post‐transfection, the best knockdown efficiency was observed in siCXCR6‐2. During 24‐72 hours after transfection, siCXCR6‐2 efficiently suppressed CXCR6 expression (Figure S1), and was chosen for subsequent studies.

16HBE cells were transfected with siCXCR6‐2 or siNC, and then treated with CXCL16 (100 ng/mL) and p38 inhibitor SB203580 (1 μmol/L)/DMSO after 24 hours. CXCL16‐stimulated epithelial barrier dysfunction was partially abolished by siCXCR6‐2 and SB203580 (Figure 3B). Consistent with the above findings, siCXCR6‐2 and SB203580 also suppressed the effects of CXCL16 on p‐p38 and E‐cadherin expression (Figure 3C). Collectively, these data indicate that CXCL16 may play a critical role in maintaining the integrity of the bronchial epithelial barrier via CXCR6 and p38 signalling.

### 3.4 LPS promotes the secretion of CXCL16 and increases the intracellular expression of CXCL16/CXCR6

**Figure 4:**
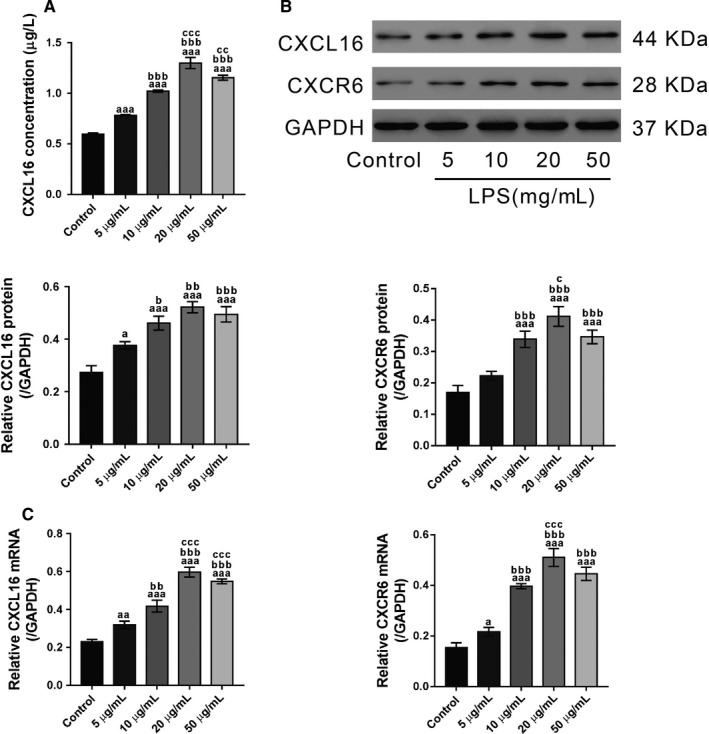
LPS promotes the secretion of CXCL16 and the intracellular expression of CXCL16/CXCR6. 16HBE cells were exposed to LPS (5, 10, 20 and 50 μg/mL) for 24 h, and control cells did not receive any treatment. CXCL16 concentration in the supernatant (A) and the protein (B) and mRNA (C) (^a^ *P* < 0.05, ^aa^ *P* < 0.01, ^aaa^ *P* < 0.001 vs control cells; ^b^ *P* < 0.05, ^bb^ *P* < 0.01, ^bbb^ *P* < 0.001 vs cells treated with 5 μg/mL LPS; ^c^ *P* < 0.05, ^cc^ *P* < 0.01, ^ccc^ *P* < 0.001 vs cells treated with 10 μg/mL LPS)

Lipopolysaccharide‐treated 16HBE cells were used as a cell model to explore the pathogenesis of ALI in vitro. To this end, 16HBE cells were exposed to different doses of LPS (0, 5, 10, 20 and 50 μg/mL) for 24 hours. Then the supernatant was collected for ELISA analysis. The results showed that the secretion of CXCL16 was increased in LPS‐exposed cells (Figure 4A). Immunoblotting (Figure 4B) and real‐time PCR (Figure 4C) analysis revealed that LPS induced a significant increase in intracellular CXCL16 at both the protein and mRNA levels, respectively. These data indicated that LPS increased both intracellular and extracellular CXCL16 expression in 16HBE cells. In addition, the intracellular protein and mRNA expression of CXCR6, the receptor of CXCL16, was also induced by LPS exposure. The effects of LPS were dose dependent from 5 to 20 μg/mL. The secretion of CXCL16 and the intracellular expression of CXCL16/CXCR6 in cells exposed to 50 μg/mL LPS were slighter lower than those in cells exposed to 20 μg/mL LPS. Therefore, 20 μg/mL was used for subsequent experiments.

### 3.5 **LPS‐mediated CXCL16/CXCR6 pathway is dependent on NF‐**κ**B**

**Figure 5:**
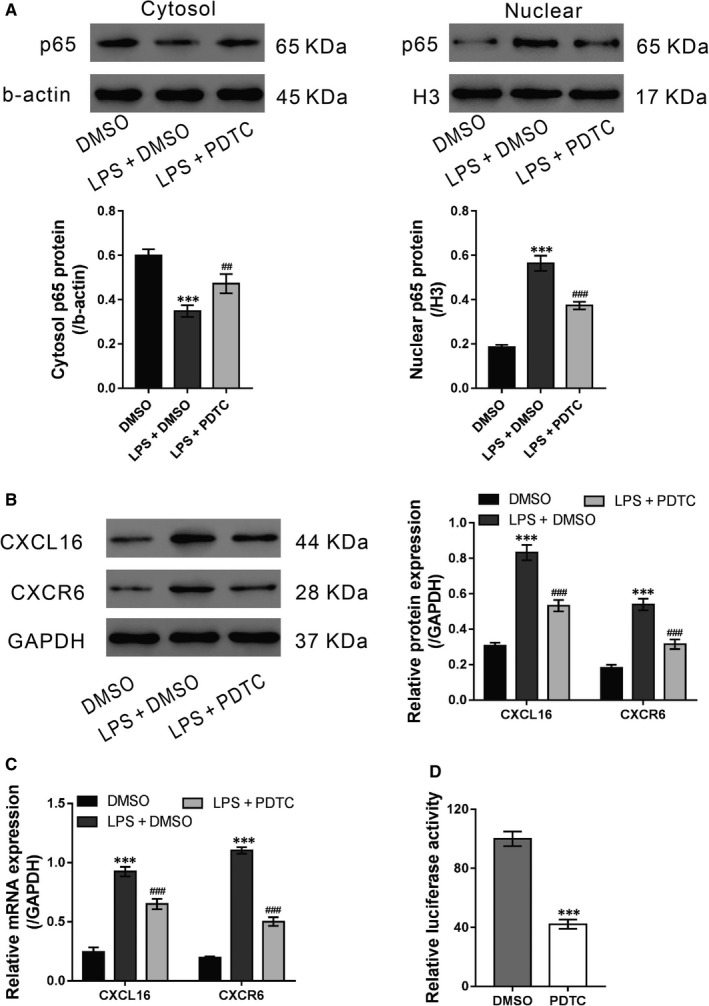
LPS‐mediated CXCL16/CXCR6 pathway was dependent on NF‐κB. A‐C, 16HBE cells were exposed to LPS (20 μg/mL) and NF‐κB inhibitor PDTC (10 μmol/L)/vehicle (DMSO) for 24 h. Cytosol and nuclear NF‐κB p65 (A, β‐actin and H3 as loading controls, respectively) and the protein (B) and mRNA (C) levels of CXCL16/CXCR6 were detected. D, Luciferase assays were performed in 16HBE cells treated with PDTC or DMSO (***P < 0.001 vs DMSO; ^##^ *P* < 0.05, ^###^ *P* < 0.001 vs LPS + DMSO)

Nuclear factor‐κB plays an important role in ALI and activates the expression of chemokines.^26^ Thus, we aimed to determine whether NF‐κB is involved in the LPS‐mediated induction of CXCL16/CXCR6. 16HBE cells were exposed to LPS (20 μg/mL) and the NF‐κB inhibitor PDTC (10 μmol/L) for 24 hours. The vehicle DMSO was used as a negative control. As shown in Figure 5A, LPS stimulation significantly increased the nuclear translocation of the p65 subunit of NF‐κB, which was suppressed by additional PDTC treatment. Moreover, PDTC treatment markedly inhibited LPS‐induced intracellular expression of CXCL16/CXCR6 (Figure 5B and 5). The results of the luciferase assay revealed that the CXCL16 promoter activity was significantly decreased by PDTC (Figure 5D), suggesting a critical role of NF‐κB on CXCL16 transcription. These data suggest that NF‐κB activation is required for the LPS‐mediated induction of the CXCL16/CXCR6 pathway.

### 3.6 CXCR6 knockout or SB203580 treatment inhibits LPS‐induced ALI in vivo

**Figure 6:**
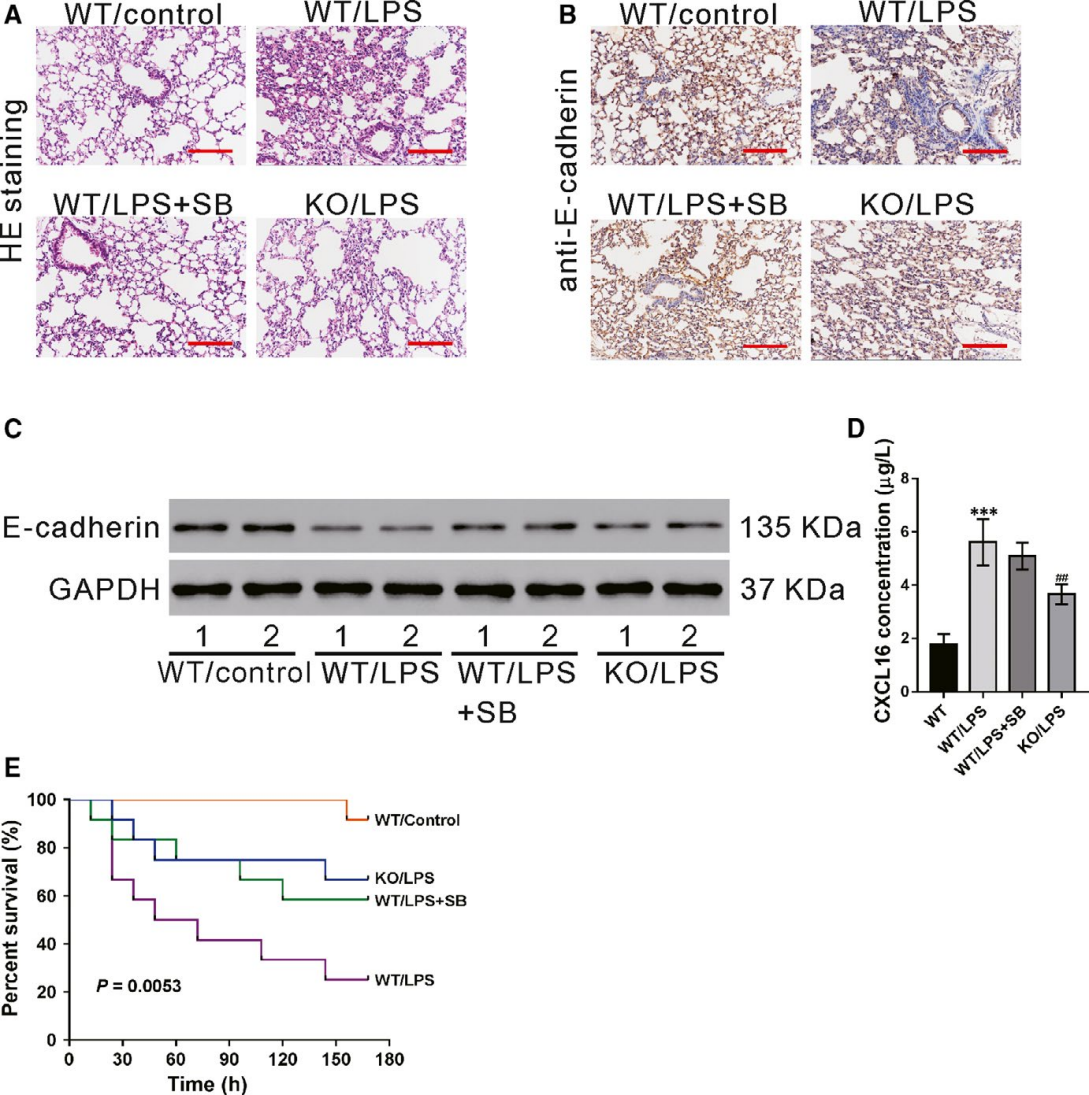
CXCR6 knockout or SB203580 treatment inhibits LPS‐induced ALI in vivo. LPS‐induced lung injury model was established in knockout (n = 5, KO/LPS group) and WT mice (n = 5, WT/LPS group). Mice in the WT/control group (n = 5) received 0.9% NaCl. Mice in the WT/LPS + SB group (n = 5) were pretreated with SB203580 and then with LPS. HE staining (A), E‐cadherin immunohistochemistry staining (B), western blotting results of E‐cadherin (C), and CXCL16 concentration in the serum (D) are shown (***P < 0.001 vs WT; ^##^ *P* < 0.001 vs WT/LPS). E, The Kaplan‐Meier plot of survival duration are shown (n = 12)

We investigated the role of CXCL16/CXCR6 in a widely used animal model of ALI.^32^, ^33^ Pathology changes in the lung tissues were determined by HE staining at 24 hours after LPS injection. As shown in Figure 6A, the normal lung structure was observed in the WT group. In contrast, the lung tissues of the WT/LPS group showed massive inflammatory cell infiltration, thickening of the alveolar wall and alveolar damage. These LPS‐induced pathological changes in the lung were markedly alleviated by SB203580 pretreatment (WT/LPS + SB group) or CXCR6 knockout (KO/LPS group). The protein levels of E‐cadherin in the lung tissues were determined by immunohistochemistry (Figure 6B) and western blotting (Figure 6C). The results showed that LPS treatment in WT mice significantly decreased E‐cadherin expression, which was partially rescued by pre‐treatment with SB203580. E‐cadherin protein levels were higher in the KO/LPS group than in the WT/LPS group. In WT mice, LPS treatment significantly promoted the secretion of CXCL16 in the serum, which was not affected by pretreatment with SB203580. The secretion of CXCL16 was lower in LPS‐treated CXCR6 knockout mice than in LPS‐treated WT mice (Figure 6D).The 7‐day survival analysis indicated that the mice in WT/LPS group had the lowest overall survival rate, while SB203580 treatment (WT/LPS + SB group) or CXCR6 knockout (KO/LPS group)markedly increased the survival rate(Figure 6E).

## 4 DISCUSSION

It is well‐known that chemokines play a key role in the pathogenesis of ALI. In this study, we first reported that CXCL16, a chemokine expressed in soluble and transmembrane forms, is elevated in the serum of ALI patients. Then, we used in vitro and in vivo experiments to explore the role of CXCL16 in epithelial barrier dysfunction and the underlying mechanisms.

First, CXCL16 treatment led to a clear decrease in the epithelial barrier integrity of the 16HBE cells. Because airway barriers prevent the inhalation of environmental factors^27^ and the disruption of epithelial barriers is involved in the pathogenesis of ALI, we speculated that elevated CXCL16 may contribute to ALI by inducing epithelial barrier dysfunction. CXCR6 knockdown significantly reversed the effects of CXCL16, suggesting that CXCL16 exerts its functions via its receptor CXCR6, which was consistent with previous observations.^22^

Excessive ROS production has been observed in patients with ALI/ARDS.^5^, ^6^, ^7^, ^8^, ^9^ Here, CXCL16 treatment increased ROS production and p38 phosphorylation in 16HBE cells. These findings are consistent with a link between ROS and p38 signalling.^39^, ^40^ E‐cadherin, a central component in the cell‐cell AJs, plays a key role in maintaining epithelial barrier integrity.^28^, ^29^, ^30^ In this study, CXCL16‐induced disruption of the epithelial barrier was associated with the loss of E‐cadherin expression. We also explored the mechanisms by which CXCL16 regulates E‐cadherin. Previous studies have demonstrated that excessive ROS can mediate the disruption of cellular AJs.^36^, ^37^, ^38^, ^41^ For example, exposure to epidermal growth factor increased the production of hydrogen peroxide in human ovarian cancer cells, which down‐regulated E‐cadherin expression through p38 activation.^41^ Here, p38 inhibitor treatment reversed the effects of CXCL16 on the epithelial barrier integrity and E‐cadherin expression. These data suggest that CXCL16/CXCR6 probably plays a critical role in E‐cadherin expression and bronchial epithelial barrier integrity via p38 signalling, although the detailed mechanisms need further investigation.

Lipopolysaccharide may induce neutrophil accumulation, ROS production and cytokine secretion, ultimately leading to ALI/ARDS.^31^ By exposing 16HBE cells to LPS, we found that LPS could enhance the release of CXCL16, and increase the expression of CXCL16 and CXCR6 at both the transcriptional and translational levels. NF‐κB, a transcription factor involved in ALI, activates the expression of chemokines.^26^ Here, the NF‐κB inhibitor PDTC significantly inhibited the effects of LPS that induced nuclear translocation of the p65 subunit and the transcription of CXCL16. Luciferase assays further demonstrated that PDTC treatment notably inhibited the activity of CXCL16 promoter. These results suggest that the mechanism underlying elevated serum levels of CXCL16 in ALI patients may involve NF‐κB activation.

In addition, the in vivo ALI model experiments showed that CXCR6 knockout or inhibition of p38 signalling might protect mice from LPS‐induced lung injury by increasing E‐cadherin expression, suggesting the therapeutic value of CXCL16/CXCR6 in ALI.

## 5 CONCLUSIONS

In conclusion, the serum levels of CXCL16 were increased in ALI patients. CXCL16 was also shown to play a critical role in decreasing E‐cadherin expression and epithelial barrier dysfunction via CXCR6 and p38 signalling. Moreover, LPS was found to enhance the transcription of CXCL16 via NF‐κB.

## CONFLICT OF INTEREST

The authors declare no competing interests.

## AUTHORS' CONTRIBUTIONS

Guo‐wei Tu, Yi‐jun Zheng and Min‐jie Ju drafted the manuscript. Li‐ming Lu and Zhe Luo conceived the proposal, revised the manuscript and provided funding support. Guang‐wei Hao performed literature searches and revised the manuscript. Guo‐guang Ma, Jun‐yi Hou and Xue‐peng Zhang performed the experiments.
