# Supplementary figures and images for: CXCL16/CXCR6 is involved in LPS‐induced acute lung injury via P38 signalling
Source: J Cell Mol Med. 2019 Jun 14;23(8):5380–9. doi: 10.1111/jcmm.14419 (PMC6653424; doi:10.1111/jcmm.14419)

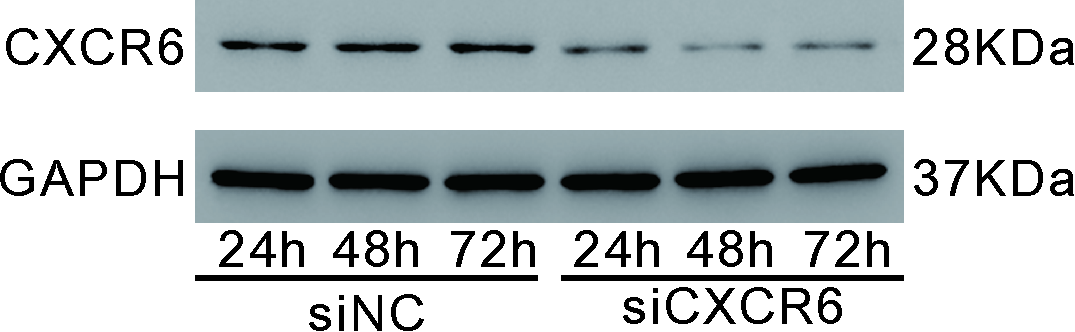

Supplement: Supplementary file 1 [file JCMM-23-5380-s001.tif]
